# The Classical Complement Pathway Is Required to Control *Borrelia burgdorferi* Levels During Experimental Infection

**DOI:** 10.3389/fimmu.2018.00959

**Published:** 2018-05-07

**Authors:** Hui Zhi, Jialei Xie, Jon T. Skare

**Affiliations:** Department of Microbial Pathogenesis and Immunology, College of Medicine, Texas A&M Health Science Center, Bryan, TX, United States

**Keywords:** spirochetes, *Borrelia burgdorferi*, complement system proteins, innate immunity, C1q

## Abstract

Activation of the classical complement pathway occurs to varying degrees within strains of the *Borrelia burgdorferi sensu lato* complex, which contain a group of pathogenic spirochetes that cause tick-borne Lyme borreliosis, including the agent of Lyme disease in the United States, *B. burgdorferi*. Despite this information, details related to the control of *B. burgdorferi* by the classical pathway are not clear. To address this question, we infected C1qα^−/−^ mice, which cannot assemble the C1 complex and thus fail to activate the classical pathway, with *B. burgdorferi sensu stricto* strain B31. Using bioluminescent *in vivo* imaging, we found that C1qα^−/−^ mice harbored more *B. burgdorferi* following 10 days of infection relative to their isogenic C57BL/6 parent. Quantitative PCR (qPCR) demonstrated that C1qα^−/−^ mice harbored significantly more *B. burgdorferi* than parent mice did within lymph nodes, skin, heart, and joints. The increased *B. burgdorferi* load in C1qα^−/−^ mice was observed at 21 and 28 days of infection, consistent with the classical pathway promoting complement-dependent, antibody-mediated killing following the development of a *B. burgdorferi*-specific humoral immune response. In addition, circulating borrelial-specific IgM was higher in C1qα^−/−^ mice relative to their parent mouse strain and did not decrease at 21 and 28 days post-infection, indicating that IgG class switching was delayed in C1qα^−/−^ mice. At day 28, both *Borrelia*-specific IgG1 and IgG3 levels were higher in infected C1qα^−/−^ mice, but that these antibodies were not sufficient to control borrelial infection in the absence of the classical pathway. Furthermore, the lack of C1q also altered the balance of the Th1/Th2 response, as both circulating Th1 (MIP-1α, IL-2, IL-12, and TNFα), Th2 (IL-4, IL-10, and MCP-1), and Th17 (IL-17) cytokines were elevated in infected C1qα^−/−^ mice. These data imply that C1q and the classical pathway play important roles in controlling borrelial infection *via* antibody and complement-dependent killing, as well as altering both antibody maturation processes and the T cell response following exposure to infectious *B. burgdorferi*.

## Introduction

Lyme disease is associated with infection following exposure to spirochetal bacteria of the *Borrelia burgdorferi sensu lato* complex, which is predominantly defined by *B. burgdorferi sensu stricto* (referred to as *B. burgdorferi* herein), *Borrelia afzelii*, and *Borrelia garinii*. These *Borrelia* species are all transmitted to human hosts through the bite of an infected tick. In the United States, *B. burgdorferi* is the leading tick-borne infection with approximately 300,000 diagnosed cases each year (1). Following infection, *B. burgdorferi* disseminates to distant organs including joints, heart, and the central nervous system (2–5). In the absence of antibiotic treatment, *B. burgdorferi* infection may lead to significant morbidity that may manifest in the form of carditis, arthritis, or neurologic pathologies. During the infectious process, *B. burgdorferi* avoids clearance *via* the innate immune system that includes complement-mediated killing. Despite this inherent resistance to innate immune mechanisms, there is much that is unknown regarding how *B. burgdorferi* persists in the face of a potent innate and adaptive immune response.

The complement system is a proteolytic cascade that interfaces with both the innate and adaptive immune responses (6–9). Following infection, the complement system can be activated by three different pathways, defined as the classical, lectin, and alternative pathways (APs), *via* the recognition of distinct microbial molecular patterns. The classical pathway is activated by the recognition of either antigen–antibody immune complexes or antibody-independent ligands *via* the globular head of C1q, while glycol ligands activate the lectin pathways. The AP is activated when surface attached C3b interacts with the protease factor B (fB) and D (fD), which results in the formation of the AP C3 convertase C3bBb (10). The AP depends on low level, yet continuous, C3b deposition by soluble C3(H_2_O)Bb convertases that occur from interactions of fB and fD with spontaneously hydrolyzed C3 “tick-over” (11). Then, AP C3 convertases amplify C3 conversion on the target surface as C3b serves the scaffold for assembly of new C3bBb convertases (12). All three pathways converge at the cleavage of complement component C3 into the anaphylatoxin C3a and opsonic C3b fragment, with the latter deposited on the microbial surface resulting in the formation of the membrane attacking complex required to lyse the cell.

Activation of the classical pathway is well known for mediating antibody-dependent clearance of microbes as C1q, together with C1r and C1s, recognizes and binds to antigen–antibody complexes with high affinity to activate the complement cascade, resulting in the formation of the membrane attack complex (MAC) and the killing of the invading organisms (13). As a part of C1, the classical complement pathway initiation complex C1qC1r_2_C1s_2_, C1q recognizes multiple Fc regions of IgM or IgG within the immune complex and enables auto-activation of the serine protease C1r, which leads to the cleavage of serine protease C1s. Activated C1s then cleaves C4 and C2 to generate the classical complement convertase C4b2b and the C5 convertase C4b2b3b, which promotes the formation of the MAC that mediates antibody-dependent killing. Within this process, the chemotactic peptides C3a and C5a are released to recruit immune effector cells to the site of infection and opsonize the pathogen through C3b, iC3b, C1q, and C4b (14).

In addition to its role in antigen–antibody recognition, various roles of C1q independent of complement activation have been observed in regulating both the innate and adaptive immune response. In the absence of C1r and C1s, immobilized C1q enhanced FcγR-mediated phagocytosis, when targets were coated with a sub-optimal concentration of antibody (15). These data suggest that C1q may aid clearance independent of antibody production in the early stages of the humoral immune response or, alternatively, in immunocompromised hosts with limited antibody production (14). C1q also decreases pro-inflammatory cytokine production and promotes the release of anti-inflammatory cytokines in macrophages, dendritic cells, and microglia, consistent with its role in the clearance of cell debris (16). In this case, C1q bound apoptotic cells are ingested by human macrophages resulting in the secretion of IL-10 and IL-27, thereby creating a less inflammatory state (17). The resulting C1q-coated apoptotic cells substantially decrease Th1 and Th17 subset proliferation and stimulate Treg proliferation, suggesting that this anti-inflammatory function of C1q skews the adaptive immune response toward a more regulatory state (18). These complement activation-independent functions of C1q may affect *B. burgdorferi* infection by regulating these different aspects of the immune response that contribute to the pathology observed. Interestingly, it has been shown that antigen-specific antibody production is delayed during borrelial infection (19). This could then result in insufficient C4b deposition on follicular dendritic cells following borrelial infection, leading to the premature collapse of germinal center and diminished antigen presentation by the follicular dendritic cells to B cells within the germinal center (20). These studies, as well as others, make it clear that, in addition to antigen–antibody recognition and classical complement activation with subsequent killing, there are additional functions associated with C1q that may be important in the host response against foreign invaders (14).

*Borrelia burgdorferi sensu lato* isolates vary in their abilities to avoid complement killing. For example, *B. afzelii* strain Pko and *B. burgdorferi* strain B31 are resistant to complement-dependent killing in normal human serum, while some *B. garinii* strains are sensitive to C1q-mediated complement killing *in vitro* since the depletion of C1q eliminated killing (21). These observations suggest that serum-resistant strains of *Borrelia* have evolved to avoid complement-dependent killing, including those associated with the classical pathway. Indeed, direct inhibitors of the classical pathway have recently been discovered in *B. burgdorferi* and include surface lipoproteins BBK32 and OspC (22, 23). While BBK32 recognizes the initiator protease C1r and inhibits its proteolytic activity, OspC binds to the complement component C4b and restricts killing *via* the classical and lectin pathways (22, 23).

Despite extensive *in vitro* studies, the function of the complement system in controlling borrelial infection *in vivo* remains unresolved, particularly in regard to the classical pathway. In this study, we compared the infectivity of wild-type *B. burgdorferi* B31 derivative strain in C57BL/6 and isogenic C1qα^−/−^ mice that are unable to activate the classical pathway to determine how the classical complement pathway affects *B. burgdorferi* infection. By using *in vivo* imaging and quantitative PCR (qPCR), along with multiplex analysis, we found that C1q plays an important role in limiting *B. burgdorferi* levels in different tissues following infection, and also affects the regulation of both cytokine and immunoglobulin production that are presumed to limit borrelial burden and resolve localized inflammation.

## Materials and Methods

### Bacterial Strains

*Borrelia burgdorferi* B31 MSK5 and ML23/pBBE22*luc* (24, 25) were grown in BSK-II media supplemented with 6% normal rabbit serum (Pel-Freez Biologicals, Rogers, AR, USA). *B. burgdorferi* cultures were grown at 32°C, 1% CO_2_, pH 7.6. ML23/pBBE22*luc* was grown with kanamycin at 300 µg ml^−1^ to provide selective pressure to maintain pBBE22*luc*. The use of infectious *B. burgdorferi* in this study was reviewed and approved by the Institutional Biosafety Committee at Texas A&M University.

### Mouse Strains

C57BL/6 parent mice were obtained from Charles Rivers. C1qα knockout mice [in the C57BL/6 background (26)] were kindly provided by Dr. Yi Xu, Texas A&M Institute for Biosciences and Technology, Houston, TX, USA. The C1qα^−/−^ mice were bred within the Texas A&M University Health Science Center vivarium facility.

### Infectivity Studies and Bioluminescent Imaging

Infectivity studies were performed as previously described (27). Briefly, 8- to 10-week-old C57BL/6 (parent) and C1qα^−/−^ mice were inoculated intradermally with 10^4^ of either *B. burgdorferi* strains MSK5 or ML23/pBBE22*luc*. For each dose and strain used, four to five mice were infected. Imaging of infected mice to detect bioluminescent *B. burgdorferi* (e.g., ML23/pBBE22*luc*) was done as previously described (25). After 10, 21, and 28 days, the mice were sacrificed and inguinal lymph node (LN), skin, heart, spleen, bladder, and tibiotarsal joint tissues were aseptically collected for *in vitro* cultivation and for qPCR analysis to quantify *B. burgdorferi* burden as described (25, 28). All animal experiments were performed in accordance to the Association for Assessment and Accreditation of Laboratory Animal Care guidelines. The Texas A&M University Institutional Animal Care and Use Committee reviewed and approved all animal procedures used in this study.

### DNA Extraction of *B. burgdorferi* from Infected Tissues and qPCR Analysis

Total DNA was isolated from mice skin, LN, heart, and tibiotarsal joint samples using the Roche High Pure PCR template preparation kit as previously described (28). Approximately 100 ng of total DNA was used for each qPCR reaction. Quantitative PCR analysis was conducted using the Applied Biosystems ABI 7900 HT system. *B. burgdorferi* genome copies and mammalian cell equivalents were determined using either the oligonucleotides nTM17FRecA (5′-GTGGATCTATTGTATTAGATGAGGCT-3′) and nTM17RRecA (5′-GCCAAAGTTCTGCAACATTAACACCT-3′) (28, 29), and primer set beta-actin-F (5′-AGAGGGAAATCGTGCGTGAC-3′) and beta-actin-R (5′-CAATAGTGATGACCTGGCCGT-3′) (30), respectively. The bacterial burden was depicted as the number of *B. burgdorferi recA* copies per 10^6^ beta-actin copies.

### Serum Collection and Multiplex Cytokine Profiling

Blood was collected by cardiac puncture in endotoxin-free tubes with no additive. The blood samples were allowed to clot at room temperature for 30 min before centrifugation (400 × *g*, 4°C, 10 min) and the serum was collected and stored at −80°C until use.

Serum cytokine levels were measured by using the Luminex 200 multiplex assay (Bio-Rad, Hercules, CA, USA), according to the manufacturer’s protocol, with a customized MilliplexMap mouse cytokine/chemokine magnetic bead panel (Millipore). Briefly, serum samples were diluted 1:2 with assay buffer, and 25 µl of bead working solution was added to each well containing either the diluted serum samples or a standard, followed by incubation overnight at 4°C with agitation. The next day, the plate was washed twice with 200 µl of wash buffer, 25 µl of detection antibody cocktail were then added to each well, and the samples incubated at room temperature for 1 h with agitation. Subsequently 25 µl of streptavidin–phycoerythrin was added to each well and incubated at room temperature for 30 min. Following an additional two washes, 150 µl of sheath fluid was added to each well. Sample data were then analyzed and calculated based on a standard curve of each analytes using the Bio-Plex Manager software. Analytes measured included IL-2, IL-4, IL-6, IL-10, IL-12, IL-17, TNF-α, keratinocyte-derived chemokine (KC), monocyte chemoattractant protein (MCP-1/CCL2), and macrophage inflammatory protein (MIP-1α/CCL3).

### Total Immunoglobulin Profiling

Serum samples were collected as mentioned earlier. Total serum immunoglobulin concentration of IgA, IgG1, IgG2a, IgG2b, IgG3, and IgM were determined using the Luminex 200 multiplex assay (Bio-Rad, Hercules, CA, USA), using a mouse immunoglobulin isotyping panel (Millipore) according to the manufacturer’s suggested protocol.

### *Borrelia*-Specific Immunoglobulin Profiling

Serum was assayed using an ELISA to quantify *B. burgdorferi*-specific antibody production using the mouse Type Isotyping kit (Bio-Rad). To conduct this assay, 96-well microtiter plates were coated with sonicated *B. burgdorferi* strain B31 at 5 µg/ml in carbonate buffer (pH 9.3), and one empty column (with no antigen) on each plate was reserved for serial dilution of purified mouse IgG1, IgG2a, IgG2b, IgG3, or IgM (eBioscience) to generate a standard curve. The plate was then blocked with 1% BSA for 1 h at room temperature. After a brief wash in PBS/0.2% Tween-20, serial dilutions of serum samples were added to the sonicate-coated wells and incubated for 1 h at room temperature. Following six washes in PBS/0.2% Tween-20, rabbit anti-mouse IgG1, IgG2a, IgG2b, IgG3, or IgM (Bio-Rad) was added to each well (as appropriate) and incubated for 1 h. Each well was then washed six times in PBS/0.2% Tween-20 and then incubated in PBS/0.2% Tween-20 containing a 1:3,000 dilution of horseradish peroxidase conjugated anti-rabbit IgG for 1 h. The wells were then washed in PBS/0.2% Tween-20, after which 3,3′,5,5′-tetramethylbenzidine was added as substrate. The enzymatic reaction was stopped after 1 min using 0.16 M sulfuric acid (Thermo Scientific), and the absorbance at 450 nm was determined. Ig content was measured by comparison to the standard curve generated from serially diluted purified Ig on individual plates.

### Data and Statistical Analysis

For qPCR analysis, a one-tailed Mann–Whitney’s *t*-test was performed between the mouse strains indicated. For the bioluminescent samples, two-way analyses of variance were performed among variables, and Bonferroni method was used to correct *P*-values for multiple comparisons. Multiple *t*-test was performed for both the cytokine and immunoglobulin profiling analyses, and the Sidak–Bonferroni method was used to correct *P*-values for multiple comparisons. Statistical significance was accepted when the *P*-values were less than 0.05 for all statistical analyses employed.

## Results

### C1q Is Required to Manage *B. burgdorferi* Infection

**Figure 1:**
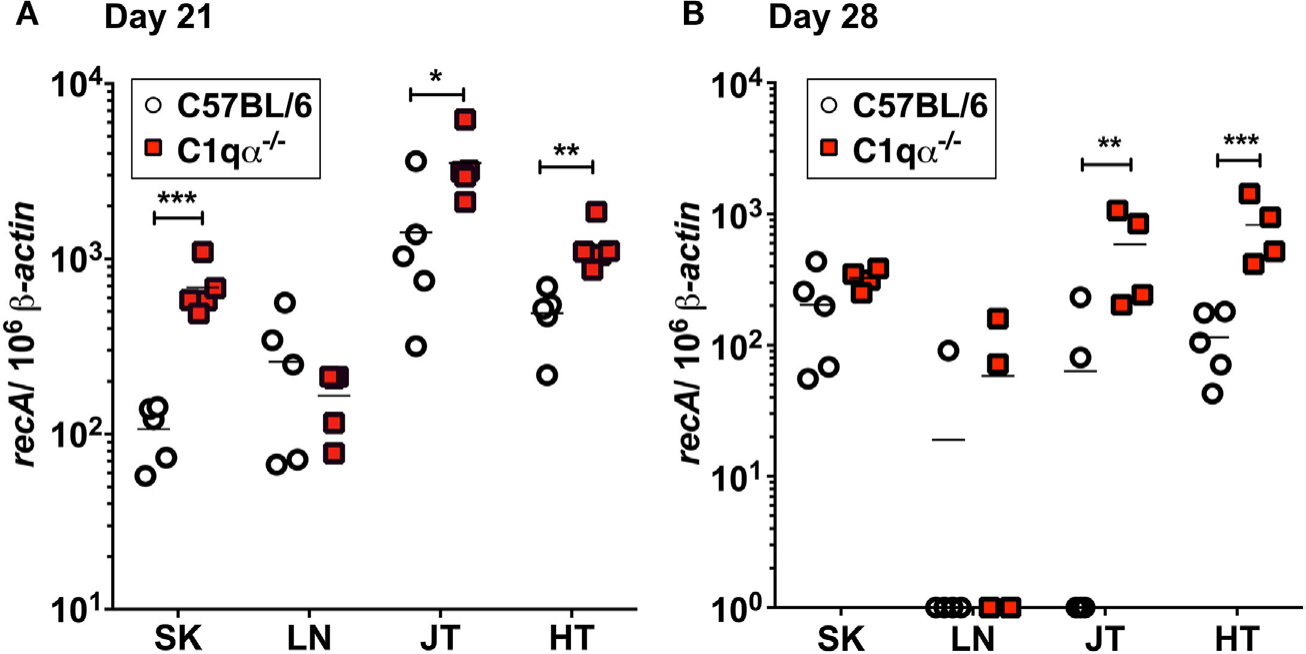
Quantitative PCR of infectious *Borrelia burgdorferi* in the parent C57BL/6 mice versus mice lacking the complement protein C1q (C1qα^−/−^). Mice were infected with 10^4^ *B. burgdorferi* strain MSK5 (24) for **(A)** 21 days and **(B)** 28 days and total DNA isolated from skin (SK), lymph node (LN), joints (JT), and heart (HT). The white circles refer to the parent C57BL/6 mouse background samples whereas the red squares indicate the values determined from the C1qα^−/−^ knockout mouse tissues samples (*n* = 4 or 5). The results are represented as the number of borrelial genome copies per 10^6^ mouse β-actin copies (*P < 0.05; **P < 0.01; and ***P < 0.001).

To determine the role of the classical complement pathway in *B. burgdorferi* infection, we inoculated C1qα^−/−^ and the isogenic parent C57BL/6 mice with 10^4^ cells of infectious *B. burgdorferi* strain B31. Borrelial burden was determined by quantitative PCR (qPCR) from skin, LN, tibiotarsal joint, and heart from C57BL/6 and C1q knockout mice at 21 and 28 days post-infection. At day 21, C1qα^−/−^ mice exhibited significantly higher bacterial load in the skin, joint, and heart, when compared with the C57BL/6 parent mice (Figure 1A). On day 28, the absolute numbers of *B. burgdorferi* were somewhat lower than that seen in the same tissues as day 21, regardless of the C1q content in the mice, most likely due to the borrelial-specific antibody killing that helps to limit the infection (Figure 1B). However, C1qα^−/−^ mice still harbored sevenfold to ninefold higher *B. burgdorferi* burden in both joint and heart tissues relative to C57BL/6 mice (Figure 1B). Taken together, these data suggest that the classical complement pathway controls systemic colonization of *B. burgdorferi*. This result is consistent with the role of classical complement function in the selective antibody-mediated killing of *B. burgdorferi*.

### The Classical Complement Pathway Limits *B. burgdorferi* Dissemination in Mice

**Figure 2:**
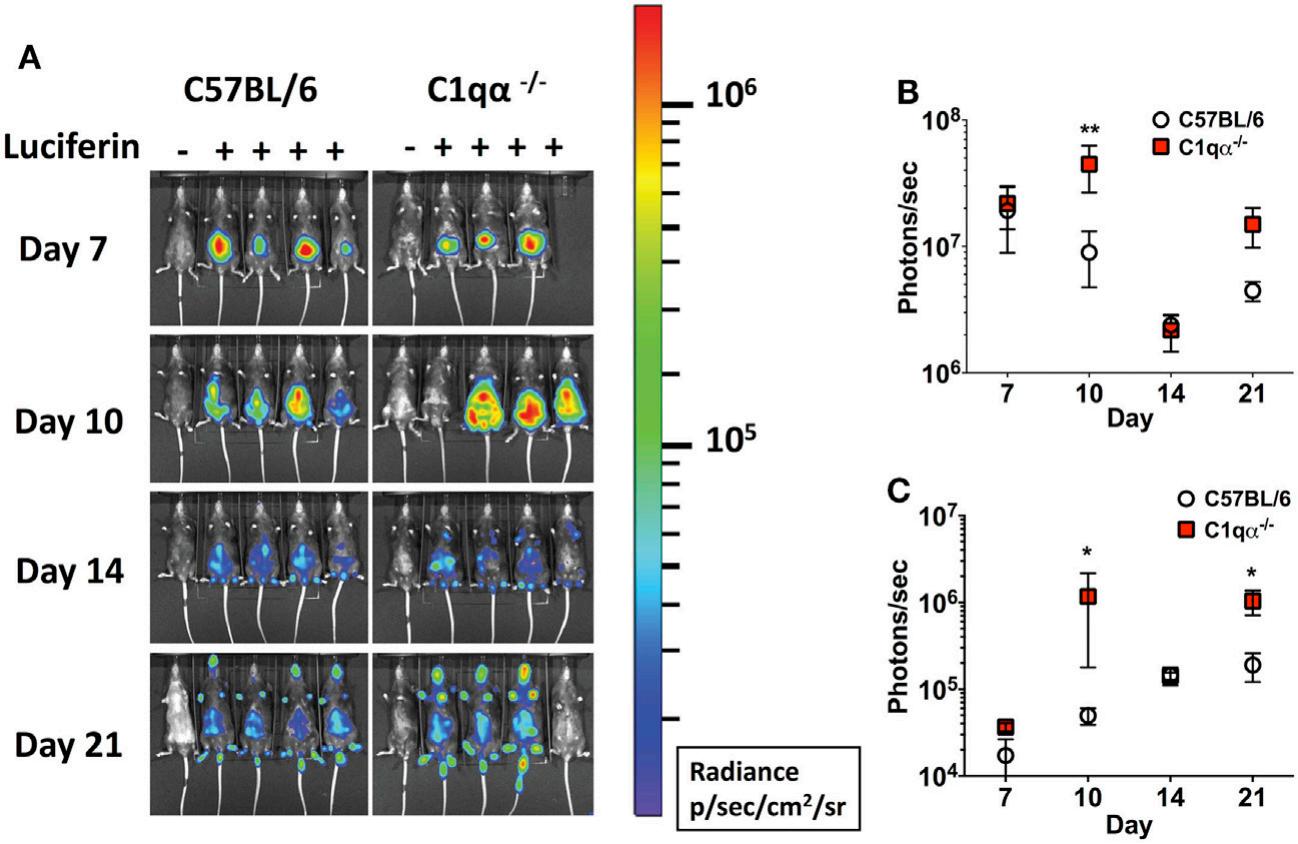
Temporal and spatial tracking of *Borrelia burgdorferi* strains following infection in C1qα^−/−^ mice. **(A)** C57BL/6 and C1qα^−/−^ mice were infected with *B. burgdorferi* containing firefly luciferase at a dose of 10^4^. Mice were treated with d-luciferin at 7, 10, 14, and 21 days post-infection and imaged. For each image shown, the mouse on the far left was infected with *B. burgdorferi* but did not receive d-luciferin serves as a background control. The image shown was obtained from a 10 min exposure. All images from each time point were normalized to the same photon/second (p/s) range of 1.15 × 10^4^ to 1.86 × 10^6^ and displayed on the same color spectrum scale (right). **(B)** Quantification of *in vivo* luminescence from each mouse. Total detected light was measured for each individual mouse in its entirety, and the total flux value was determined (in photons/second). The total flux values from mice treated with d-luciferin were normalized by subtracting the background total flux value of the infected mouse with no added luciferin relative to mice that were given luciferin. Total flux was determined after infection at days 7, 10, 14, and 21 for mice infected with 10^4^ *B. burgdorferi*. **(C)** Quantification of *in vivo* luminescence from the joint region. The joint localized signal was selectively measured, and the total flux value was determined (in photons/second). Each data point represents the average signal from the left and right ankles of each mouse. White circles and red squares represent signal from C57BL/6 and C1qα^−/−^ mice, respectively, both infected with ML23/pBBE22*luc* (*n* = 3 or 4; *P < 0.05 and **P < 0.01).

Next, we asked if the classical complement pathway controls *B. burgdorferi* infection at early stage of infection before borrelial-specific IgG develops, by tracking the active infection using *in vivo* imaging. To this end, we infected the C57BL/6 parent and C1qα^−/−^ mice with a B31 derivative strain containing borrelial codon-optimized firefly luciferase (ML23/pBBE22*luc*) (25) at dose of 10^4^ cells and measured light emission from the live bacteria following exposure to d-luciferin substrate. At early time points, e.g., 1 h, 1, 4, and 7 days post-infection, C57BL/6 and C1qα^−/−^ mice demonstrated equivalent light emission from skin tissue (data not shown), suggesting that C1q does not alter the innate immune response to control borrelial replication and colonization. At day 7, the light signal peaked in C57BL/6 mice and started to decline at day 10, suggesting the bacterial load had peaked following 1 week of infection. In C1qα^−/−^ mice, *B. burgdorferi* levels reached their maximum intensity at day 10 (Figure 2A) at a level that was approximately fourfold higher than signal detected in C57BL/6 mice when all detectable light from each mouse is compared (Figure 2B). Light emission from the joint area was also approximately twofold higher in C1qα^−/−^ mice than relative to the C57BL/6 parent at day 10 of infection (Figure 2C). It is important to note that we cannot rule out the possibility that light emission from joints at day 10 was partially due to signal originating from the overlying skin. It is likely that the classical pathway is activated by antigen-specific IgM at day 10 and kills *B. burgdorferi* in an IgM-dependent manner. Interestingly, although the colonization of *B. burgdorferi* in both C57BL/6 and C1qα^−/−^ mice decreased to a similar level at day 14, light emission is almost fivefold higher at day 21 in the joints of all C1qα^−/−^ mice relative to the joints of the C57BL/6 parent mice (Figure 2C), suggesting that the classical complement pathway controls *B. burgdorferi* levels at secondary colonization sites. Another possibility is that antibody-independent C1q-mediated opsonization occurs *via* macrophages that clear *B. burgdorferi* (31). Given that there is little difference observed in clearance before day 10 for the parent or C1qα^−/−^ mice, it seems less likely that the differences observed are due to macrophage clearance but instead are the result of C1q-mediated antibody-dependent killing either *via* MAC formation or delayed opsonization by neutrophils following the development of *B. burgdorferi*-specific antibodies (31).

### C1q-Deficient Mice Accumulate Significantly Higher *B. burgdorferi* Following Dissemination

**Figure 3:**
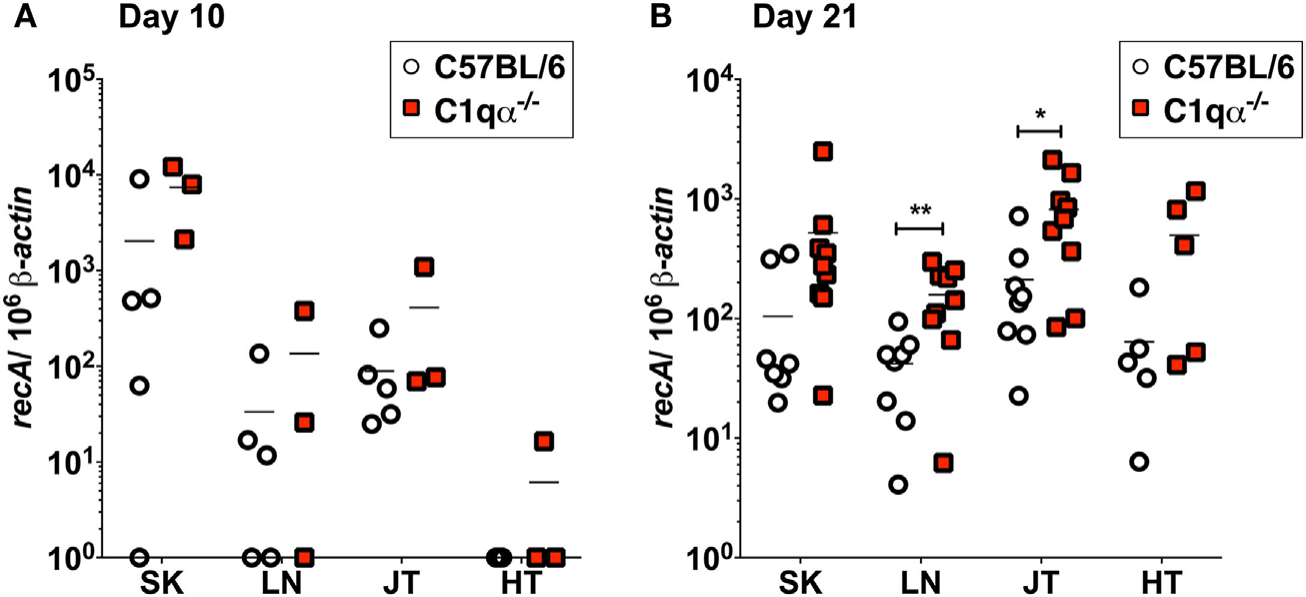
Bacterial load of *Borrelia burgdorferi* in C57BL/6 and C1qα^−/−^ mice. Quantitative PCR (qPCR) from mice infected with 10^4^ *B. burgdorferi* strain (ML23/pBBE22*luc)* following 10 days **(A)** or 21 days **(B)**. White circles and red squares indicate the data points from samples of C57BL/6 and C1qα^−/−^ knockout mice, respectively [*n* = 3–4 for panel **(A)**; *n* = 9 for panel **(B)**]. Abbreviations: SK, skin; LN, lymph node; JT, joints; HT, heart. The results are represented as the number of borrelial genome copies per 10^6^ mouse β-actin copies (*P < 0.05 and **P < 0.01).

To quantify *B. burgdorferi* colonization in deeper tissues of C57BL/6 and C1qα^−/−^ infected mice at an earlier time point, quantitative PCR (qPCR) was performed to enumerate spirochete levels in skin, LN, tibiotarsal joint, and heart at day 10 relative to 21 days post-infection. At day 10, more bacteria were recovered from the skin of C1qα^−/−^ mice than the C57BL/6 parent (Figure 3A); however, the differences observed were not statistically significant due to the large variation seen in the C57BL/6 mice. These data are consistent with the *in vivo* imaging result on day 10 (Figure 2A). In contrast to skin samples, *B. burgdorferi* was present at low levels in LNs, joint, and heart tissues (Figure 3A), suggesting that *B. burgdorferi* had poorly colonized these sites at day 10. At day 21, a higher *B. burgdorferi* load was detected from all tested tissues from C1qα^−/−^ mice relative to the C57BL/6 parent (Figure 3B), a result that is consistent with the increased borrelial load for MSK5 in Figure 1A and the higher light signal observed for the luciferase-producing *B. burgdorferi* (Figure 2).

### Absence of C1q Alters Cytokine/Chemokine Levels in Response to *B. burgdorferi* Infection

**Figure 4:**
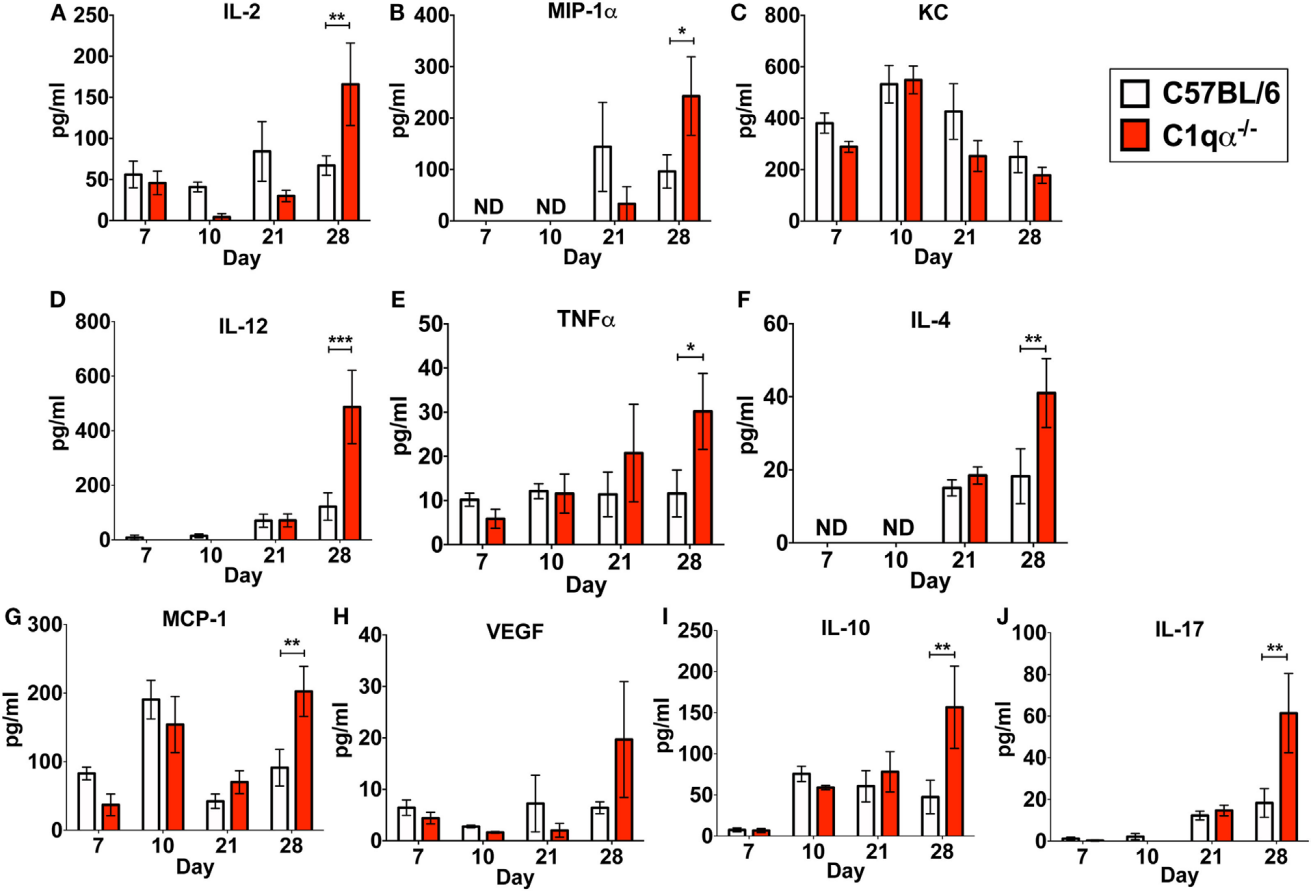
C1q deficiency affects the level of circulating cytokines in infected mice. Whole blood was collected from mice infected with 10^4^ *Borrelia burgdorferi* strain MSK5 (24) at 7, 10, 21, and 28 days post-infection. Serum was isolated by centrifugation, and the level of 10 circulating cytokines **(A–J)** was determined using the Bio-Plex system employing the Luminex multianalyte profiling technology. The white column represents C57BL/6 mice, whereas the red column refers to data associated with C1qα^−/−^ mice; *n* = 4–5 for samples from days 7, 10, and 21; *n* = 10 for samples from day 28. Abbreviation: ND, not detected (*P < 0.05; **P < 0.01; and ***P < 0.001).

In addition to its role in the classical complement pathway, C1q also affects the inflammatory response following and concurrent with infections and tissue damage (32, 33). To determine if the absence of C1q alters the inflammatory response following *B. burgdorferi* infection, we measured the levels IL-2, IL-4, IL-10, IL-12p70, IL-17, KC, MCP-1, MIP-1α, TNFα, and VEGF in sera derived from C57BL/6 parent and C1qα^−/−^ mice infected with *B. burgdorferi* at 7, 10, 21, and 28 days post-infection. None of the cytokines tested showed significant different level of production between C57BL/6 and C1qα^−/−^ mice at days 7, 10, and 21 (Figure 4). However, at day 28, levels of IL-2, IL-4, IL-10, IL-12, IL-17, MCP-1, MIP-1α, and TNFα were significantly higher in C1qα^−/−^ relative to the C57BL/6 parent mice (Figure 4). More specifically, IL-2, IL-10, IL-12, and IL-17 increased approximately threefold, while IL-4, MCP-1, and MIP-1α increased twofold in mice lacking C1q (Figure 4). This result indicates that C1q affects the regulation and/or balance of the Th1 (MIP-1α, IL-2, IL-12, and TNFα), Th2 (IL-4, IL-10, and MCP-1), and Th17 (IL-17) responses to *B. burgdorferi* infection. However, the imbalance of T cell response observed in C1qα^−/−^ mice was restricted in *B. burgdorferi*-infected mice, as cytokine response was not observed in the mock-infected C57BL/6 parent or C1qα^−/−^ mice (data not shown).

### Lack of C1q Alters Immunoglobulin Subtype Population During Borrelial Infection

The importance of antibody production in the control of *B. burgdorferi* infection has been well addressed both *in vitro* and *in vivo*. Previous studies have shown that *B. burgdorferi*-specific IgG in polyclonal antisera from experimentally infected mice and from humans with Lyme disease, as well as selected monoclonal IgG produced from infected mice, can prevent colonization in mice, but are incapable of eliminating *B. burgdorferi* from previously infected mice (34–38).

**Figure 5:**
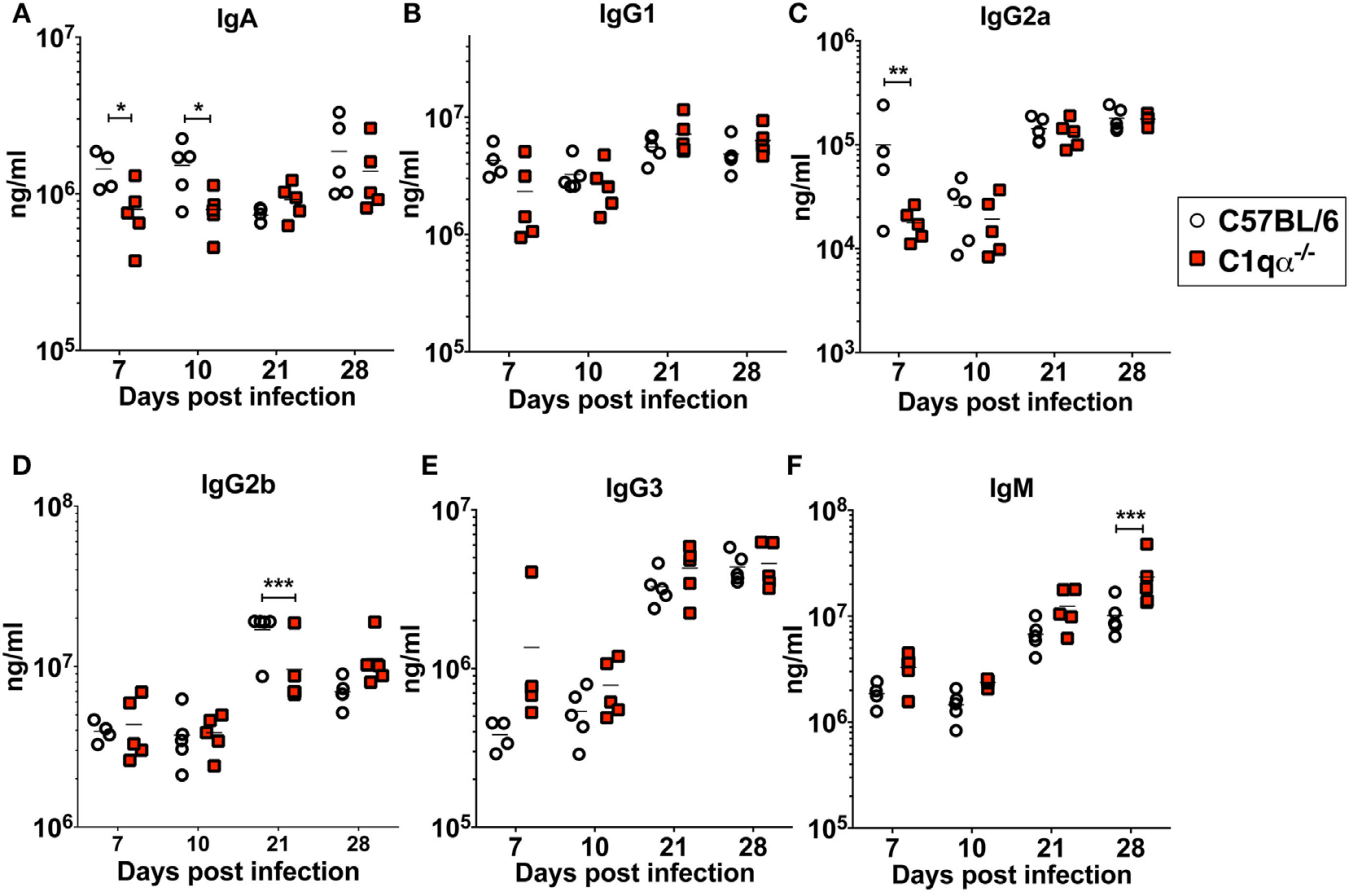
Lack of C1q affects the level of immunoglobulins IgA **(A)**, IgG1 **(B)**, IgG2a **(C)**, IgG2b **(D)**, IgG3 **(E)**, and IgM **(F)** in infected mice. Whole blood was collected from mice infected with 10^4^ *Borrelia burgdorferi* strain MSK5 (24) at 7, 10, 21, and 28 days post-infection. Serum level of immunoglobulins was determined by the Bio-Plex system employing the Luminex multianalyte profiling technology. White circles refer to samples from C57BL/6 mice, whereas red squares represent data from C1qα^−/−^ mice (*n* = 5; *P < 0.05; **P < 0.01; and ***P < 0.001).

To determine if the lack of the classical complement pathway affects antibody development during borrelial infection, we measured the levels of each subtype of total immunoglobulins using a multiplex approach, and *B. burgdorferi*-specific immunoglobulins IgA, IgG, and IgM in sera from C57BL/6 and C1qα^−/−^ mice infected with *B. burgdorferi* using an ELISA based assay (Figures 5A–F). The results showed that the total amount of IgG2a are lower in C1qα^−/−^ mice before day 10 (Figure 5C). At day 21 post-infection, IgG2b level is significantly lower in C1qα^−/−^ mice relative to C57BL/6 mice (Figure 5D). C1qα^−/−^ mice showed marginally higher production of IgM at days 7, 10, and 21 when compared with C57BL/6 mice, but, at day 28, the level increased significantly (Figure 5F). These data suggest that the absence of C1q may slow the development of antibody subtypes and affect IgG class switching, which may represent a contributing factor in the decreased clearance of *B. burgdorferi* observed in C1qα^−/−^ mice. However, we cannot exclude the possibility that B cells that express different immunoglobulin isotypes are selectively expanded.

**Figure 6:**
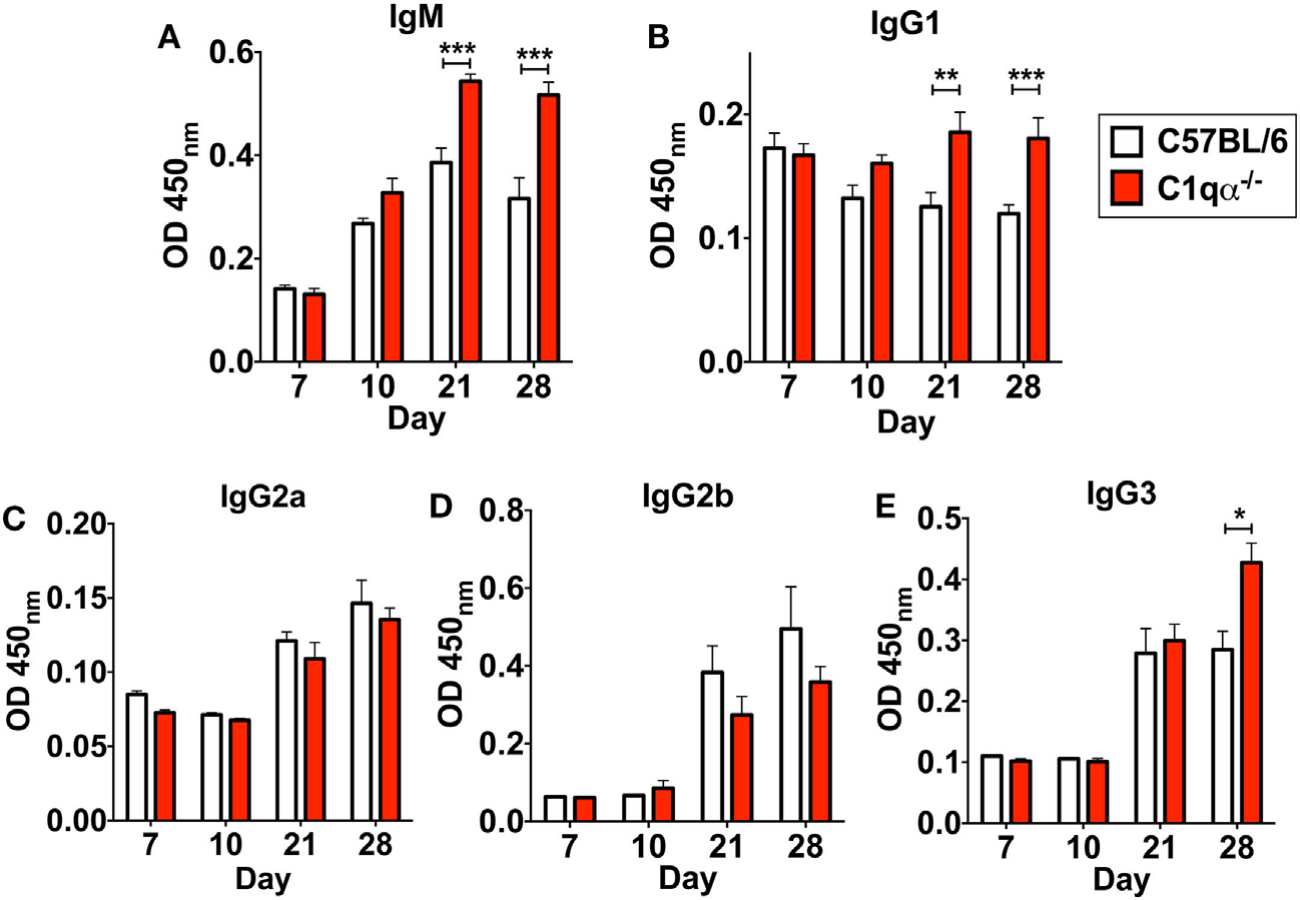
Lack of C1q alters the level of *Borrelia*-specific antibody subtypes IgM **(A)**, IgG1 **(B)**, IgG2a **(C)**, IgG2b **(D)**, and IgG3 **(E)** in infected mice. Whole blood was collected from mice infected with 10^4^ *Borrelia burgdorferi* strain MSK5 (24) at 7, 10, 21, and 28 days post-infection. Serum level of *Borrelia*-specific immunoglobulin subtypes was determined by ELISA using total protein lysate derived from *B. burgdorferi* strain MSK5. White columns refer to C57BL/6 mice, whereas red columns represent data from C1qα^−/−^ mice (*n* = 5; *P < 0.05; **P < 0.01; and ***P < 0.001).

In addition to affecting total antibody development, C1qα^−/−^ mice also developed different subtype populations of *B. burgdorferi*-specific antibodies relative to C57BL/6 mice. Here, the level of *B. burgdorferi*-specific IgM in C1qα^−/−^ mice was significantly higher than in C57BL/6 parent after day 21 and was stably maintained at day 28, whereas the level of IgM in C57BL/6 mice reached the peak at day 21 and then started to decrease at day 28 (Figure 6A), suggesting that the IgG isotype class switching was delayed in C1qα^−/−^ mice. Similarly, the level of *B. burgdorferi*-specific IgG1 and IgG3 was higher in C1qα^−/−^ mice than C57BL/6 after day 21 and 28, respectively (Figures 6B,E), whereas the level of *B. burgdorferi*-specific IgG2a and IgG2b are slightly lower in C1qα^−/−^ mice when compared with their C57BL/6 parent (Figures 6C,D). However, although C1qα^−/−^ mice produced more *B. burgdorferi*-specific IgM, IgG1, and IgG3, they still harbored higher number of bacteria than C57BL/6 at these two time points (Figure 1), suggesting that C1q is essential for clearance by *B. burgdorferi*-specific antibodies, and that the antibodies alone fail to manage borrelial infection.

## Discussion

*Borrelia burgdorferi sensu lato* isolates exhibit different sensitivities to the various host complement pathways (21, 39, 40). Despite these differences, it remains controversial whether the complement system controls borrelial infection *in vivo*, and, specifically, the role of the classical complement pathway in borrelial clearance is still unclear. In this study, we evaluated the role of the classical complement pathway in the infection of *B. burgdorferi* by comparing the course of infection in mice genetically deficient in complement component C1q α-chain (C1qα^−/−^) to that of its parent, the C57BL/6 mouse. Using *in vivo* imaging, combined with qPCR quantification, we showed that C1qα^−/−^ mice harbored more *B. burgdorferi* than the isogenic parent mouse in skin tissues as early as 10 days of infection, and in skin, joint, and heart tissues at later stages of infection (Figures 1 and 3). In addition to limiting *B. burgdorferi* numbers, the absence of C1q also results in dysregulation of the Th1/Th2 cytokine and chemokine response that may affect localized tissue inflammation (Figure 4). Furthermore, the lack of C1q alters the total and borrelial-specific antibody production, which may compromise the adaptive immune response to borrelial infection and result in a higher bacterial burden. Taken together, this study demonstrates critical roles of the classical complement pathway in *B. burgdorferi* infection, not only to limit borrelial colonization by facilitating antibody killing but to also in its role in altering the development of the adaptive immune response, which may augment tissue inflammation.

Antibodies are an important adaptive immune mechanism used to control infection. In the case of *B. burgdorferi*, passive transfer of borrelial-specific antibodies quells infectivity, indicating an important role for humoral immunity in borrelial clearance (34–38). In this regard, and within the context of borrelial-specific antibody, both complement-dependent and -independent responses contribute to the clearance of *B. burgdorferi* (41, 42). In this study, we found higher bacterial burden in the skin, joint, and heart of C1qα^−/−^ mice at 3 weeks post-infection when compared with parent mice, indicating that the presence of C1q limits borrelial load, likely by promoting borrelial-specific antibody-mediated killing. After 4 weeks of infection, the bacterial numbers were similar in the skin tissues of C1qα^−/−^ and parent mice, but prominently lower than that in the skin of C1qα^−/−^ mice at day 21, suggesting that a C1q-independent antibody response to *B. burgdorferi* limited the infection in skin tissues at this stage. Interestingly, *in vivo* imaging demonstrated that the colonization of *B. burgdorferi* in both the parent and C1qα^−/−^ mice decreased to a similar level by day 14, indicating that a C1q-independent antibody against *B. burgdorfer*i likely also played a role in reducing borrelial levels at this time point (Figure 2). Earlier studies showed that borrelial-specific antibodies can be bactericidal *in vitro* independent of the presence of complement (42, 43). Regarding *in vivo* studies, Schmitz *et al*. showed that passive immunization with anti-*Borrelia* serum in hamsters was abrogated by the depletion of complement (41). However, the complement-dependent protection was only provided by serum taken from hamsters 1 and 10 weeks post-infection, whereas the serum taken at 3 weeks following infection cleared *B. burgdorferi* independent of complement, presumably due, in part, to borreliacidal antibodies to OspB (42). By contrast, our data showed that the classical pathway decreased the bacterial load at both 10 and 21 days of infection (Figure 3). It is worth noting that the aforementioned study used an inoculum of 10^6^ *B. burgdorferi*, which is 100-fold higher than the dose used here, and employed a different animal model (41). These differences could explain why the temporal and spatial dynamics of the *B. burgdorferi* infection and antibody response observed here is not in agreement with the hamster infectivity data. Nevertheless, both studies showed that complement-dependent and -independent antibodies control *B. burgdorferi* at a different stage of infection. Although the C1q-independent antibody appears to limit *B. burgdorferi* colonization in the skin by day 28, our results also show that C1qα^−/−^ mice exhibit a significant increase in borrelial burden within both joint and heart tissues, indicating that the C1q-dependent antibody clearance contributes to the control of *B. burgdorferi* in those tissues (Figure 1B).

Using *in vivo* imaging and qPCR quantification, we found that C1qα^−/−^ mice harbored more bacteria in skin tissue relative to parent mice. It is unlikely that borrelial-specific IgG controls the infection at day 10 or earlier since high titer and high affinity IgG develop only after class switching has occurred. One mechanism that might limit *B. burgdorferi* expansion early is recognition of naïve and borrelial antigen-specific IgM antibodies, which have a 1,000-fold greater binding affinity to C1q when compared with IgG (44, 45). Given that borrelial-specific IgM develops early and as prelude to class switching, it is likely that the antigen-specific IgM also controls borrelial infection in a C1q-dependent manner. This observation is consistent with prior studies demonstrating that IgM limits *B. burgdorferi* infection. Specifically, Belperron and Bockenstedt found that infected ticks feeding on B-cell-deficient mice (B6*Igh^−/−^*) harbor more spirochetes than those that fed on wild-type mice. Passive transfer of either normal mouse serum or natural mouse IgM into B6*Igh^−/−^* mice reduced spirochete burden in feeding ticks, whereas naïve IgG antibodies had no effect (45). These observations suggest that IgM is involved in both innate and adaptive immune response following borrelial infection and the data presented herein link C1q to IgM-mediated clearance. However, IgM may be more efficient in killing *B. burgdorferi* in the blood during dissemination rather than in the connective tissues where *B. burgdorferi* prefer to colonize, due to the large molecular size of IgM that prevents it from reaching *B. burgdorferi* in deeper tissues (19). This hypothesis may explain why we failed to detect any difference in borrelial colonization between C1qα^−/−^ and the parent mice before day 10. It is also likely that natural IgM-dependent killing before day 10 is independent of C1q or that the number of *B. burgdorferi* cleared by natural IgM is beyond the detection limitation of imaging and qPCR employed here.

Bacterial burden of *B. burgdorferi* is, in part, controlled by complement mediated, antibody-dependent killing *via* different mechanisms, including antibody directed killing (42), lysis of bacteria by MAC formation, and phagocytosis of opsonized spirochetes (46, 47). During natural infection with *B. burgdorferi*, phagocytic cells play a critical role in pathogen control at both early and later stages of infection. Phagocytosis of *B. burgdorferi* by activated macrophages *in vitro* can occur independent of borrelial antigen-specific antibodies (31). On the other hand, phagocytosis by polymorphonuclear leukocytes is mediated by the borrelial antigen-specific antibodies to opsonize the spirochetes (31). Therefore, the loss of C1q and differences within the immunoglobulin population in C1qα^−/−^ mice may lead to decreased phagocytosis by neutrophils, as well as less C1q-dependent MAC formation, which could contribute to the increased bacterial load in those mice. However, since the C57BL/6 parent and C1qα^−/−^ mice did not show differences in bacterial load before day 10 post-infection, we contend that the differences observed are due to C1q-dependent killing mechanisms that require the development of antibodies directed against *B. burgdorferi*.

C1q is also known to regulate T cell development and immune response (47), which is linked to joint inflammation following *B. burgdorferi* infection (48). In this study, we also asked if C1q deficiency alters the systemic immune response to *B. burgdorferi* infection, by determining the level of cytokines and chemokines in the blood of infected mice at different stages of infection. We found that Th1, Th2, and Th17 associated cytokines were all significantly higher in the C1qα^−/−^ mice only following 28 days of infection when compared with the parent mice (Figure 4), whereas the level of cytokines in mock-infected mice were not affected (data not shown). All other time points showed only modest differences (Figure 4). The simultaneous elevation of Th1, Th2, and Th17 associated cytokines was also observed in systemic lupus erythematosus patients, which is associated with C1q deficiency (49–51), suggesting C1q plays a role in balancing T cell response in tissue inflammation and damage. As indicated earlier, the significant elevation of cytokines/chemokines were only observed in C1qα^−/−^ mice infected with *B. burgdorferi* for 28 days, suggesting that the imbalance of T-cell response in C1qα^−/−^ mice involves a cell-mediated response to borrelial infection. It has been shown that C1q bound to apoptotic cells suppresses human macrophage and dendritic cell-mediated Th1 and Th17 T cell subset proliferation (52), which could explain why the lack of C1q fails to limit Th1 and Th17 response to borrelial infection, given that *B. burgdorferi* infection is purported to induce apoptosis in monocytes (53). However, it is still not clear why the Th2 response was elevated. One possibility is that inflammation mediated by Th1 and Th17 subtype also triggers stimulation of the Th2 response. It is also possible that the higher level of IL-10 produced in infected C1qα^−/−^ mice (Figure 4) stimulates anti-inflammatory Th2 response and protects *B. burgdorferi* from elimination, given that borrelial-specific IL-10 production is known to repress spirochete clearance (54, 55).

It is well established that the classical pathway is involved in antibody-dependent clearance of pathogens. In this study, we showed that C1qα^−/−^ mice harbored more *B. burgdorferi* both 21 and 28 days post-infection. One likely explanation for this observation is that the complement-mediated, antibody-dependent killing of *B. burgdorferi* is reduced in the C1qα^−/−^ mice, leading to the retention of greater numbers of the spirochetes (Figure 1). It is also possible that C1q deficiency compromises the development of both the primary and secondary antibody response, which could diminish *B. burgdorferi* clearance (56). To test this possibility, we quantified levels of six immunoglobulin sub-classes following borrelial infection and found that the total amount of IgA, IgG2a, and IgG2b are reduced in C1qα^−/−^ mice when compared with the parent strain, while IgM levels are higher in C1qα^−/−^ mice. In this regard, C1q deficiency not only compromises antibody killing, but appears to impair antibody production during the infection, both of which contribute to reduced *B. burgdorferi* antibody-mediated clearance. One additional explanation posits that the loss of C1q leads to decreased C4b deposition on follicular dendritic cells, which may cause premature collapse of germinal center response, due to diminished antigen presentation by the dendritic cells to B cells within the germinal center (20). In fact, *B. burgdorferi* infection leads to strong and sustained IgM response and delayed development of long-lived antibody and B cell memory (19, 20), perhaps due to abrogated C4b deposition.

In addition to the total antibody level, the level of *B. burgdorferi*-specific IgM, IgG1, and IgG3 are increased in C1qα^−/−^ mice relative to C57BL/6 parent, while IgG2a and IgG2b are not affected or mildly decreased (Figure 6). The increased level of *Borrelia*-specific IgM in C1qα^−/−^ mice parallels with the observations of altered B cell response during *B. burgdorferi* infection such that serum IgM levels remain high and the ratio of IgG and IgM is relatively constant (47, 57). These data suggest that the dampened B cell response during borrelial infection could be due to the delayed activation of the classical complement pathway. In fact, *B. burgdorferi* interferes with the activation of the classical pathway *via* surface proteins, such as BBK32 and OspC (22, 23, 58). Furthermore, interference of the classical pathway activation could also contribute to *B. burgdorferi* persistence in immunocompetent hosts with the presence of borrelial-specific antibody. In addition to IgM, the level of IgG1 and IgG3 are also higher in C1qα^−/−^ mice. These three isotypes all bind to C1q and activate the classical pathway, whereas IgG2a and IgG2b bind to the Fc receptor (56). As such, it is likely that those immuno-complexes formed with C1q-binding antibodies cannot be opsonized by the complement system during infection due to the absence of C1q, thus fail to be engulfed by phagocytes and accumulated within the circulation system.

In summary, we demonstrate here that the classical complement pathway controls *B. burgdorferi* infection *in vivo*, in part by facilitating borrelial-specific antibody-dependent killing of the bacteria. In addition, we find that both complement-dependent and -independent antibody killing play a role in limiting *B. burgdorferi* numbers at different stages of infection. Our study also demonstrates that C1q affects the cytokine production related to both T cell and B cell responses in borrelial infection, both of which could control borrelial burden and resolve tissue inflammation. Further studies are warranted to investigate whether other borrelial proteins interact with C1q to determine whether *B. burgdorferi* employs additional strategies to neutralize the classical complement pathway to promote survival in the mammalian hosts they infect.

## Ethics Statement

All animal experiments were performed in accordance to the Association for Assessment and Accreditation of Laboratory Animal Care (AAALAC) guidelines. The Texas A&M University Institutional Animal Care and Use Committee (IACUC) reviewed and approved all animal procedures/protocols used in this study.

## Author Contributions

HZ and JS outlined the research plan and wrote the manuscript. HZ, JX, and JS conducted the experimentation and evaluated the data obtained.

## Conflict of Interest Statement

The authors declare that the research was conducted in the absence of any commercial or financial relationships that could be construed as a potential conflict of interest.
